# Cell Migration Research Based on Organ-on-Chip-Related Approaches

**DOI:** 10.3390/mi8110324

**Published:** 2017-10-31

**Authors:** Xiaoou Ren, David Levin, Francis Lin

**Affiliations:** 1 Department of Physics and Astronomy, University of Manitoba, Winnipeg, MB R3T 2N2, Canada; renx34@myumanitoba.ca; 2 Department of Biosystems Engineering, University of Manitoba, Winnipeg, MB R3T 2N2, Canada; david.levin@umanitoba.ca

**Keywords:** microfluidic device, cell migration, organ-on-chip

## Abstract

Microfluidic devices have been widely used for cell migration research over the last two decades, owing to their attractive features in cellular microenvironment control and quantitative single-cell migration analysis. However, the majority of the microfluidic cell migration studies have focused on single cell types and have configured microenvironments that are greatly simplified compared with the in-vivo conditions they aspire to model. In addition, although cell migration is considered an important target for disease diagnosis and therapeutics, very few microfluidic cell migration studies involved clinical samples from patients. Therefore, more sophisticated microfluidic systems are required to model the complex in-vivo microenvironment at the tissue or organ level for cell migration studies and to explore cell migration-related clinical applications. Research in this direction that employs organ-on-chip-related approaches for cell migration analysis has been increasingly reported in recent years. In this paper, we briefly introduce the general background of cell migration and organ-on-chip research, followed by a detailed review of specific cell migration studies using organ-on-chip-related approaches, and conclude by discussing our perspectives of the challenges, opportunities and future directions.

## 1. Introduction

**Figure 1:**
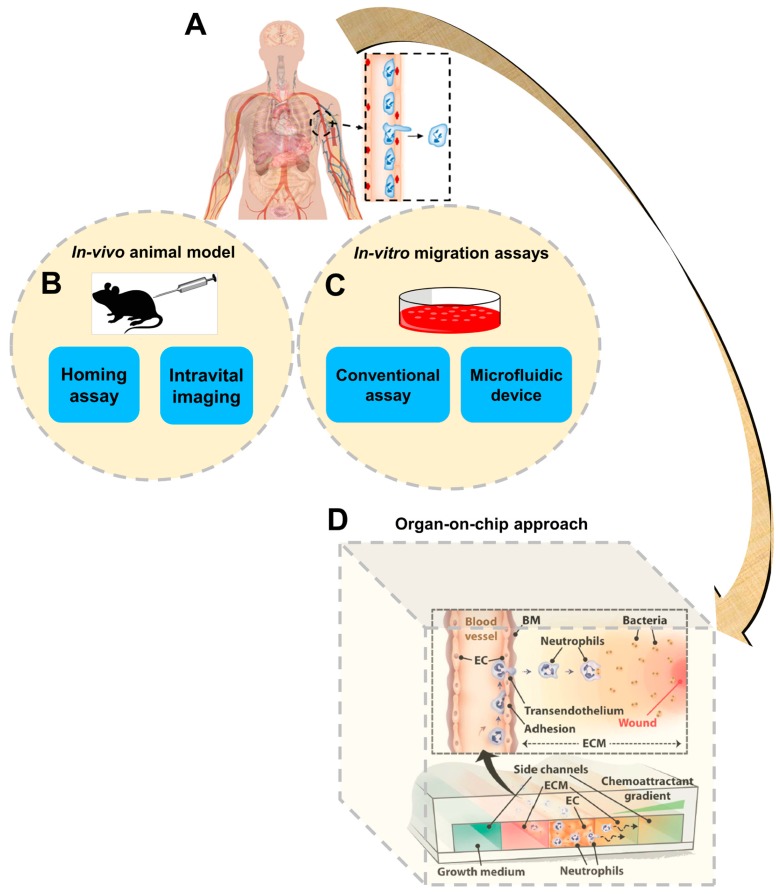
A schematic illustration of different cell migration research approaches to study this important and complicated process in humans. (**A**) Schematic presentation of human body and transendothelial migration of immune cells; (**B**) Schematic presentation of in-vivo animal models; (**C**) Schematic presentation of in-vitro cell migration assays; (**D**) An example of the organ-on-chip approach. Figure 1D was adapted from reference [35] with permission from the Royal Society of Chemistry. BM: basement membrane; EC: endothelial cell; ECM: extracellular matrices.

The physical structure of cells is supported and organized by dynamic functions of the cytoskeleton. Dynamic remodeling of this structure enables cell movements in diverse biological contexts, such as cell–cell migratory interaction, wound healing and transmigration across tissue barriers [1]. The migratory abilities of cells is critically involved in a wide range of physiological activities, including embryogenesis, homeostasis, immune responses, tissue regeneration and axon guidance, as well as cancer metastasis [2,3,4,5,6,7,8]. In tissues, cell migration is guided by a wide range of environmental cues, such as chemical gradients, electric fields and mechanical stimulations [9,10,11,12]. Sensing environmental cues that guide cell migration, such as chemoattractants, typically involves coordinated regulation by the tissue-specific adhesion molecules and chemoattractants that interact with their counter-receptors on the migrating cells, followed by further processing of the chemotactic signals by downstream signaling pathways [13,14,15]. For example, upon inflammation, target tissues upregulate the expression of specific adhesion molecules and chemoattractants, which recruit immune cell subsets expressing the corresponding homing receptors [16]. Therefore, cell migration research requires the ability to configure and control complex cellular microenvironments. In-vivo animal models, such as homing assays or intravital imaging methods, directly provide the tissue microenvironment (Figure 1), and those approaches have been widely used for cell trafficking studies. However, those approaches are expensive, involve complicated procedures and require sacrificing animals [17]. Reduction and/or replacement of animal-based research is a high priority for both the research community and the public [18]. In addition, cell migration environments are poorly defined in animal experiments and observation of cell migration in vivo is greatly limited by the current state-of-the-art imaging capability. Ultimately, the applicability of results generated by animal studies for human healthcare applications is limited by significant variations between humans and experimental animals [19,20].

In-vitro cell migration assays, such as transwell assays [21] and other real-time visualization chambers, are another broadly used research approach to study cell migration (Figure 1). Compared with animal studies, in-vitro cell migration assays are more cost-effective, easier to operate, and can directly test human cells. Nevertheless, although cell migration conditions are better defined in these assays, they are lacking the ability to precisely control the cell migration environment in space and time, and the conditions configured in these assays are over-simplified compared with the in-vivo situation.

Microfluidic devices were rapidly developed over the last two decades and these devices have been increasingly used for cell migration research (Figure 1) [22,23]. Microfluidic devices offer significant advantages in device miniaturization, precise configuration and flexible manipulation of cellular microenvironments such as stable chemical gradient generation, low reagent consumption, real-time observation of cell migration at the single-cell level and high-throughput experimentation [9,11,24,25]. On the other hand, the majority of the current microfluidic cell migration devices are only capable of producing much-simplified chemical fields and extracellular matrices (ECM) compared with tissue environments. Many of these devices focus on single cell types and ECM conditions, and thus do not capture the complex properties of tissues and organs in vivo. Additionally, although cell migration has high disease relevance and microfluidic devices have the potential to enable disease-oriented cell migration studies, very few microfluidic cell migration studies to date have involved testing clinical samples from patients to investigate the disease mechanism or for diagnostic assessment [26,27]. Therefore, there has been a growing trend to develop new microfluidic devices to better reconstitute the complex in-vivo microenvironment at the tissue or organ level, which is commonly referred to as “organ-on-chip” (Figure 1) [28,29,30].

Generally speaking, the construction of organ-on-chip systems is based on microfluidic cell-culture devices, which simulate the key activities and responses of certain tissues or organs. This approach integrates microfluidics technology with tissue engineering, permitting investigation of organ-specific physiological mechanisms and diseases. For example, some studies successfully established “liver-on-chip” and “kidney-on-chip” systems for studying hepatic or renal functions, respectively [31,32,33]. Similarly, a recent study constructed a biomimetic “skin-on-a-chip” model for drug toxicity testing and disease study [34]. Thus, organ-on-chip offers a novel and advanced research approach for cell migration study.

Indeed, in this direction, we have observed growing development of cell migration studies involving organ-on-chip-related approaches. In this paper, these studies are broadly defined as meeting one or more of the following criteria: (1) cell migration and interaction studies that involve reconstituting the physiological structures of specific organs using multi-cell co-culture in three-dimensional (3D) microfluidic models; (2) cell migration studies in microfluidic devices that configure complex chemical fields relevant to target organs; and (3) cell migration studies that test clinical cell or/and tissue samples for diagnostic assessment of organ-specific diseases. Under these selection criteria, the rest of this paper is organized to review organ-specific cell migration studies using organ-on-chip-related approaches including “tumor-on-chip”, “lung-on-chip”, “vessel-on-chip”, “lymph node (LN)-on-chip” and “brain-on-chip” (Table 1). We conclude by discussing our perspectives of the challenges, opportunities and future directions.

## 2. Tumor-on-Chip

**Figure 2:**
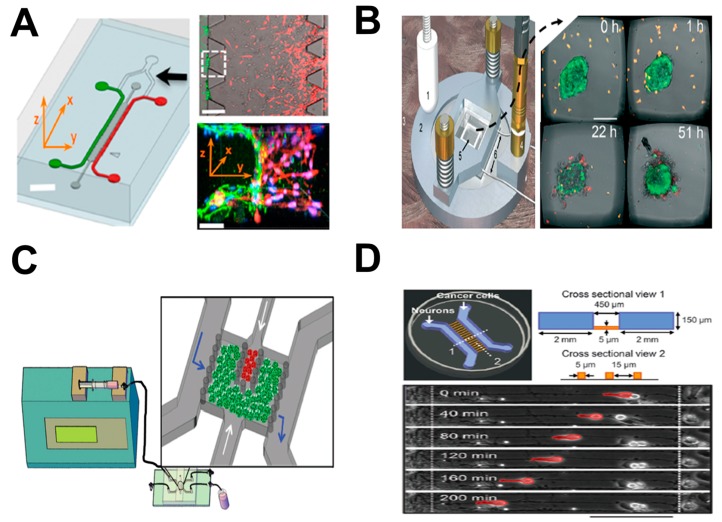
Examples of tumor-on-chip cell migration study. (**A**) The 3D microfluidic model for investigating endothelial barrier function during tumor cell intravasation. The left panel shows the microfluidic device (green: endothelial cell channel; red: tumor channel; dark gray: 3D ECM channel; black arrow: Y junction); the upper-right panel shows a representative phase-contrast image of tumor cell (red) invasion through 3D ECM region (dark gray) to the endothelium (green); the bottom-right panel shows a 3D confocal image of the selective area in the white-dashed square (red: tumor cells; green: endothelium); (**B**) The ultrasonics-based 3D microdevice for studying immune surveillance of NK cells for specific tumors. The left panel illustrates the main components of the microfluidic system; the right panel shows some representative experimental images of NK–tumor interaction at different time points (orange: NK cells; green: solid tumors); (**C**) The breast-cancer-on-chip model to investigate ECM activation during tumor progression. The figure shows the microfluidic system and magnified view of the chip (green: stromal microtissues; red: tumor microtissues; blue arrow: fluid flow direction; white arrow: 3D microtissue injection direction); (**D**) The tumor-on-chip model to investigate the interactions between neurons and cancer cells during tumor PNI. The upper panel illustrates the microfluidic device; the bottom panel shows a representative image of tumor cell (red) migration behavior along the contacted neurites at different time points. Figure 2A was adapted from reference [36] with permission from the National Academy of Sciences; Figure 2B was adapted from reference [37] with permission from the Royal Society of Chemistry; Figure 2C was adapted from reference [38] with permission from John Wiley and Sons; Figure 2D was adapted from reference [39] with permission from the Royal Society of Chemistry.

Cancer cell migration is a critical process during cancer progression and metastasis. During metastasis, tumor cells migrate from their initial locations to distant organs, leading to new tumor formation [53,54,55]. Cell–cell interaction and chemotaxis are two important biological processes that are involved in tumor metastasis [56,57]. However, the mechanism of tumor metastasis is far from being well-understood, in part hindered by the limitations of current cell migration research methods in controlling the complex physiological tumor microenvironments [58]. Organ-on-chip offers a promising new approach to tumor cell migration studies. Here we highlight some examples in this direction (Figure 2; Table 1).

As one example, Kamm and coworkers developed a three-dimensional microfluidic model for investigating endothelial barrier function during tumor cell intravasation (Figure 2A) [36]. The device consists of two side microchannels for seeding of the tumor and endothelial cells (ECs), respectively, and these two channels were connected via a middle channel that was filled with 3D hydrogel. An endothelial monolayer was formed at all the 3D ECM–endothelial interface regions and enabled the observation of transmigrating tumor cells across the vascular lumen in real time. By using this tumor-on-chip approach, the 3D physiological tumor–vascular interface was reconstituted in vitro. These results demonstrated that cellular interaction with macrophages or stimulation with tumor necrosis factor alpha (TNF-α) increased endothelial permeability, resulting in endothelial barrier impairment and a higher tumor cell intravasation rate. In another recent study, this device was used to study anti-tumor efficacy of engineered TCR-T cells [59]. This system enabled the observation and analysis of specific T cell immune surveillance, including T cell directional migration to tumors and their subsequent tumor-killing function on the chip.

As another example, Wiklund and coworkers established an ultrasonics-based 3D microdevice for studying immune surveillance of natural killer (NK) cells for specific tumors (Figure 2B) [37]. Briefly, this device was comprised of a multi-well microplate and a ring-shaped ultrasonic transducer. The microplate was made of a silicon wafer with arrayed (10 × 10) wells in the center, which was bonded onto a glass slide. This multi-well microplate was surrounded by a polydimethylsiloxane (PDMS) frame bonded to the top layer, providing a pool for cell medium injection. A cover slip was placed onto the PDMS frame in order to minimize evaporation. The ultrasonic transducer consisted of a ring-shaped piezoceramic plate and an open central hole for visualization under a microscope. By using controlled ultrasonic waves produced by the transducer under well-regulated environmental conditions, biomimetic 3D tumors composed of human hepatocellular carcinoma (HCC) HepG2 cells were achieved simultaneously in each well of the microplate. This approach enabled the observation of NK cell migration and the interactions between NK cells with 3D solid tumors. In addition, the number of NK cells required for preventing tumor growth in the first day, or destroying a tumor in the following days, was determined using this system.

In another study, Imparato and coworkers developed a breast-cancer-on-chip model to investigate ECM activation during tumor progression (Figure 2C) [38]. This model consisted of two main components: (1) a syringe pump for continuous medium perfusion and metabolic waste removal at controlled flow rate; and (2) a microfluidic chip for mimicking the breast tumor microenvironment. Briefly, the chip had one main chamber and three connected channels. The main chamber was divided into a big stromal compartment and a small tumor compartment, respectively. Both compartments were loaded with 3D microtissues (μTP). Normal fibroblast microtissues (NF-μTP) or cancer-activated fibroblast microtissues (CAF-μTP) were injected into the stromal compartment, while malignant epithelial breast cancer cell microtissues (MCF7-μTP) were injected into the tumor compartment. Based on this tumor-on-chip approach, a 3D engineered tumor microenvironment was established. This system enabled monitoring and analysis of tumor–stromal interactions and tumor–ECM communications during tumor invasion toward the adjacent stromal compartment in real time. These results demonstrated that 3D fibroblast μTP can promote tumor cell invasion resulting from increased secretion of paracrine molecules in a 3D environment compared to a 2D environment.

Finally, Jiang and coworkers developed a tumor-on-chip model to investigate the interactions between neurons and cancer cells during tumor perineural invasion (PNI) (Figure 2D) [39]. The microfluidic chip consisted of two cell-culture channels that were interconnected by parallel arrayed microgrooves, bonded onto a culture dish. The left channel and the right channel were used for loading neurons and cancer cells, respectively. Primary hippocampal and cortical neurons from embryonic Sprague-Dawley (SD) rats and dorsal root ganglion (DRG) neurons from postnatal SD rats were used in the study. These neurons were mixed and cultured in the device. In addition, three different human cancer cell lines, including a prostate cancer cell line (PC-3), a pancreatic cancer cell line (Panc-1) and a breast cancer cell line (MCF-7), were selected according to their different levels of tumor PNI [60]. Co-culture of these physiologically relevant cells in the microfluidic chip mimicked the pathological microenvironment during cancer PNI. These results demonstrated that neurites facilitated cancer cell attachment and induced their directional migration. Moreover, cancer cells with high PNI levels (PC-3 and Panc-1) exhibited stronger migratory behavior along neurites than cancer cells with low PNI levels (MCF-7). Thus, interruption of neurites and blockade of the neuron–cancer interaction has the potential to inhibit cancer cell migration.

## 3. Lung-on-Chip

**Figure 3:**
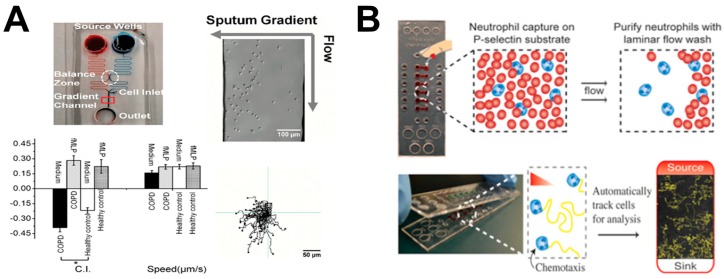
Examples of lung-on-chip cell migration study. (**A**) Microfluidics-based approach for investigating neutrophil chemotaxis with clinical samples for rapid diagnosis of COPD. The upper-left panel illustrates the microfluidic device; the upper-right panel shows a representative cell image in the device; the bottom-left panel shows cell migration test data using the microfluidic device (C.I.: chemotactic index); the bottom-right panel shows cell tracks from a representative experiment; (**B**) Microfluidics-based approach for investigating neutrophil chemotaxis with clinical samples for asthma detection. The upper panel illustrates rapid on-chip neutrophil isolation from blood; the bottom panel illustrates the microfluidic method for neutrophil chemotaxis test. Figure 3A was adapted from reference [26] with permission from PLOS; Figure 3B was reproduced from reference [27] with permission from the National Academy of Sciences. fMLP: *N*-formyl-methionyl-leucyl-phenylalanine.

Neutrophils are the largest population among white blood cells, and their migration plays important roles in the human immune system [61]. Disordered neutrophil migration and recruitment can result in pulmonary inflammation and associated lung diseases, such as asthma and chronic obstructive pulmonary disease (COPD) [62,63]. Organ-on-chip technologies provide a useful approach to study cell migration in the mimicked lung microenvironment. In addition, testing cell migration using clinical samples from patients with lung diseases offers useful cell functional diagnostic assays. Here we review some examples of organ-on-chip cell migration studies related to either mimicking the lung microenvironment or pulmonary disease diagnosis (Figure 3; Table 1).

Ingber and coworkers engineered a biomimetic lung-on-chip microsystem, which replicated the alveolar–capillary structures of human lung, for investigating bacteria or inflammatory cytokine-induced cell migration. The microfluidic device consisted of two side chambers, and one main channel with two compartments separated by a porous ECM-coated PDMS membrane [40]. The upper compartment was an air-filled channel, which was used for seeding lung alveolar epithelial cells. The lower compartment was a microvascular channel, which was used for lining lung microvascular ECs. The mimicked human breathing activities were achieved by applying mechanical strain rhythmically from the side chambers. The integration of rhythmical mechanical stretching in the side chambers, cyclic air–liquid interflow within the membrane and regulated fluid flow in the microvascular channel enabled the patterning of epithelial cells and ECs onto the membrane for differentiation. This “breathing” lung-on-chip model enabled the investigation of lung-specific immune response to bacteria- or cytokine-induced inflammatory processes. In order to mimic pulmonary inflammation, TNF-α and Escherichia coli (*E. coli)* bacteria were injected into the alveolar epithelial cell layer, which rapidly activated ECs and increased the surface expression of intercellular adhesion molecule 1 (ICAM-1) in the microvascular channel. The results showed that this effect induced neutrophil recruitment to ECs, and stimulated their directional transmigration across the tissue barrier for immune surveillance. This system demonstrated a low-cost on-chip model for cell migration study and provided an alternative option to animal models for drug delivery and toxicity research.

Instead of mimicking the lung microenvironment, other studies focused on testing immune cell migration using clinical samples from patients with specific lung diseases for potential diagnostic applications. COPD is a type of lung disease associated with breathing difficulty, which is caused by narrowed airways [64]. A previous study had demonstrated the correlation between COPD and neutrophil chemotactic infiltration to the airways [65]. Thus, neutrophil chemotaxis has the potential to characterize and diagnose COPD. In this direction, Lin and coworkers developed a microfluidic device to study neutrophil chemotaxis to the supernatant gradient of sputum samples from healthy donors and COPD patients (Figure 3A) [26]. The results showed stronger neutrophil chemotaxis to the sputum of COPD patients than the sputum of healthy donors. In addition, the results confirmed the important chemical factors in COPD sputum for inducing neutrophil chemotaxis. More recently, the same group further developed an all-on-chip method for rapid analysis of neutrophil chemotaxis [41]. The novel device had a unique cell-docking structure that enabled cell alignment on one side of the gradient channel, permitting accurate and rapid chemotaxis readout without time-lapse cell tracking. In addition, neutrophils were directly isolated from a drop of blood using the on-chip magnetic separation method. In this study, a rapid and integrated neutrophil isolation and chemotaxis test to a known recombinant chemoattractant or clinical sputum from COPD patients was achieved in 25 min. These developments demonstrated the potential of a microfluidic-based cell migration test method for diagnosing inflammatory lung disease. As another example in a similar direction, Beebe and coworkers developed a microfluidic method to investigate chemotaxis of neutrophils from asthma patients for diagnostic application (Figure 3B) [27]. The device consisted of a lid part as the chemoattractant source, and a base part as the neutrophil capture sink. When the lid was placed onto the base, the chemoattractant diffused into a gradient in the microchannel to induce neutrophil chemotaxis. The results showed lower chemotaxis velocity of neutrophils in the samples from asthmatic patients compared to non-asthmatic patients, suggesting neutrophil chemotaxis can be potentially used for asthma diagnosis.

## 4. Vessel-on-Chip

**Figure 4:**
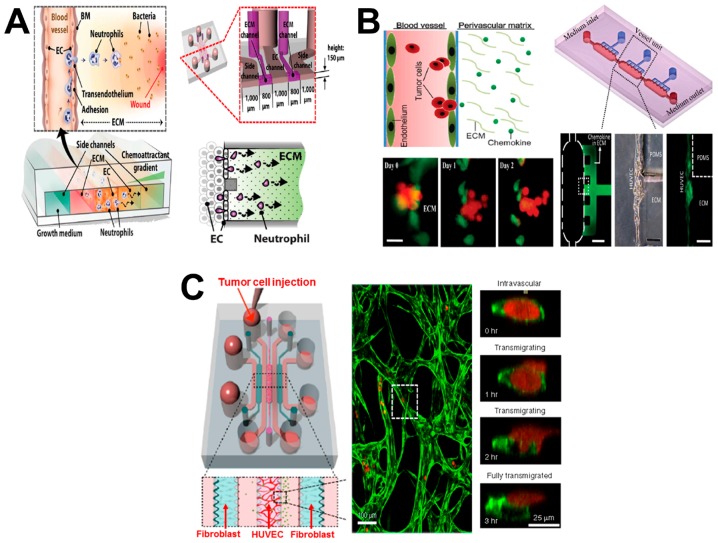
Examples of vessel-on-chip cell migration study. (**A**) The vessel-on-chip model for investigating neutrophil TEM during the inflammatory process. The left panel shows the schematic presentation of neutrophil TEM under inflammatory conditions; the upper-right panel shows the dimensions of the microfluidic device; the bottom-right panel shows the schematic presentation of neutrophil TEM (side view); (**B**) The vessel-on-chip model for investigating tumor TEI. The upper-left panel shows the schematic presentation of tumor TEI; the bottom-left panel shows representative experimental images of tumor cells (red) during TEI; the upper-right panel shows the microfluidic device; the bottom-right panel shows representative experimental images of the selective area; (**C**) The vessel-on-chip model for investigating tumor cell extravasation. The left panel shows the microfluidic device and the detailed information of the selective area; the middle panel shows the magnified view of the selective area (green: microvascular network; red: tumor cells); the right panel shows representative experimental images of one transmigrating tumor cell in the white dashed box. Figure 4A was adapted from reference [35] with permission from the Royal Society of Chemistry; Figure 4B was adapted from reference [42] with permission from the Royal Society of Chemistry; Figure 4C was adapted from reference [43] with permission from Nature Publishing Group. PDMS: polydimethylsiloxane.

Leukocyte transendothelial migration (TEM) from blood vessels to inflammatory sites is a critical process for human immune responses. This migration process is mediated by both physical and chemical cues through complex interactions between leukocytes and ECs [66,67]. Similarly, the transendothelial invasion (TEI) of cancer cells through blood vessels to target tissues (i.e., tumor extravasation) is an important process during tumor metastasis [58,68]. These relevant research topics require more advanced microfluidic systems to configure the complex in-vivo-like vascular microenvironment for investigating different cell types involved in transvascular migration (TVM) during various pathological processes. Here we describe some examples of vessel-on-chip for TVM and angiogenesis studies (Figure 4; Table 1)

Chung and coworkers developed a vessel-on-chip model for investigating neutrophil TEM during inflammatory processes (Figure 4A) [35]. The microfluidic device consisted of five connected channels, including two side channels, two ECM channels and one EC channel. The ECM layer and EC monolayer patterning were achieved by collagen injection and EC seeding within the ECM channels and EC channel, respectively. The EC channel was loaded with neutrophils, and side channels were injected with growth medium and chemoattractants, reconstituting in-vivo TEM under multiple inflammatory stimulations. Different concentrations of *N*-formyl-methionyl-leucyl-phenylalanine (fMLP) and interleukin-8 (IL-8) were tested for inducing neutrophil chemotaxis. In addition, the number and migration behavior (e.g., distance and velocity) of neutrophils that transmigrated through both the EC monolayer and ECM layer were measured. These results showed that fMLP gradients more strongly attracted neutrophils than IL-8 gradients, and that neutrophil–EC interactions were indispensable to neutrophil TEM during the inflammation process. Despite its utility in the cell migration study, this system can be used as a potential disease model for drug screening as well.

In another study, Qin and coworkers developed a biomimetic blood-vessel-on-chip model for investigating tumor TEI (Figure 4B) [42]. The model reconstituted the primary features of physiological blood vessels, including vessel lumen, endothelium and perivascular chemokine-containing ECM. The main channel connected with medium inlet and outlet and was filled with cancer cells and culture medium, serving as the lumen of blood vessels. The side channels connected to the main channel and matrix inlet and were filled with cultrex basement membrane extract (BME) and chemokines, mimicking the perivascular ECM. In addition, human umbilical vein endothelial cells (HUVECs) were seeded onto the BME surface to mimic endothelium. Based on this model, salivary gland adenoid cystic carcinoma (ACC) cell TEI was recorded and analyzed in real time under a well-controlled physiological microenvironment. The results demonstrated ACC TEI was induced by a C-X-C Motif Chemokine Ligand 12 (CXCL12) gradient, resulting in irreversible impairment of endothelial integrity. However, AMD 3100, an efficient CXCR4 antagonist, was able to inhibit this invasive process, but not inhibit the adhesion of ACC to the endothelium. This vessel-on-chip approach showed its feasibility for vasculature modeling and its potential for tumor TEI investigation.

As another example, Kamm and coworkers established a microvasculature-on-chip model for investigating tumor cell extravasation (Figure 4C) [43]. Briefly, the microfluidic device consisted of three hydrogel lumens, separated by medium channels in between, allowing chemical factor delivery and exchange. HUVECs–hydrogel and fibroblasts (FBs)–hydrogel mixtures were loaded into the middle and side hydrogel regions, respectively. The boundary between each hydrogel lumen and medium channel was made of an array of microposts that allow cell–hydrogel mixture expansion and paracrine interactions of HUVECs and FBs, simultaneously. After microvascular network (μVN) formation, tumor cells were injected through the HUVEC gel region for real-time observation. This vessel-on-chip model enabled multiple on-chip cell culture, mimicking of human vasculature reconstitution, and single cell tracking, allowing investigation of tumor TEM and micrometastases formation.

Besides these TVM-oriented studies, Neumann and coworkers developed a microvessel-on-chip model for investigating angiogenesis [44]. The device was made of two bonded PDMS stamps, embedded with two microfibers in between. The two parallel microchannels were interconnected by a main chamber. The chamber was injected by the mixture of collagen gel and human vascular pericytes. After gel formation, the microfibers were removed, and the top microchannel was perfused with growth factors, while HUVECs were introduced into the bottom microchannel. This model reconstituted the 3D features of in-vivo vasculature, including tubular vessel structure, multiple cell co-culture, mimicked ECM substrate and controlled chemical microenvironment. Based on this vessel-on-chip model, the angiogenesis process was studied by observing directional migration of pericytes toward the growth factor gradient. This approach provided a new method for organotypic vasculature establishment, which facilitated studying cell migration related vascular functions.

## 5. Lymph Node (LN)-on-Chip

**Figure 5:**
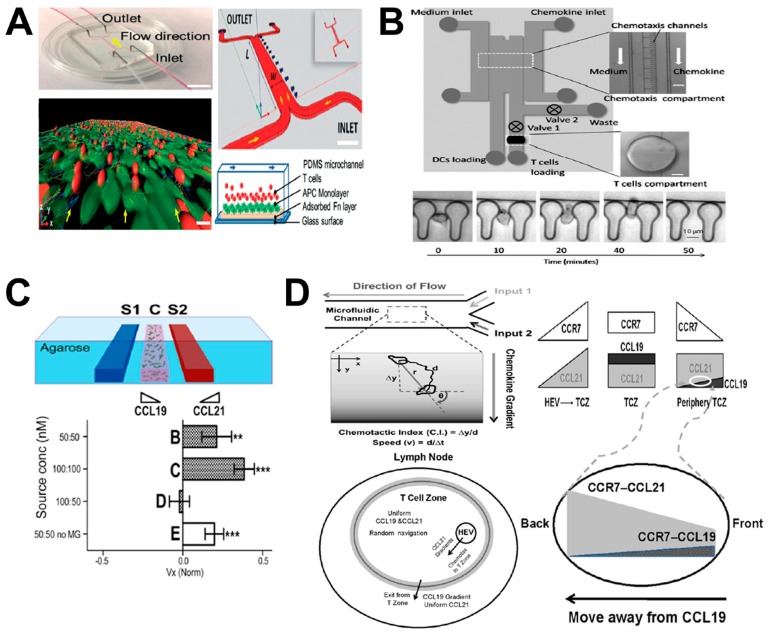
Examples of LN-on-chip cell migration study. (**A**) The LN-on-chip flow device for investigating the interaction between T cells and DCs. The upper-left and upper-right panels show the real microfluidic device and its schematic illustration, respectively; the bottom-left panel shows one representative 3D confocal image of the interaction between T cells (red) and DC monolayer (green); the bottom-right panel shows the schematic illustration of the microchannel in side view; (**B**) The LN-on-chip model for evaluating DC chemotaxis and DC–T cell interaction. The upper panel shows the microfluidic device; the bottom panel shows representative data of CCL19 gradient-induced mature DC (mDC) migration. (**C**) The 3D agarose-based microfluidic device for investigating differential chemotaxis of DCs to CCL21 and CCL19. The upper panel shows the schematic illustration of microfluidic device (side view) (S1&S2: chemokine/buffer loading channels; C: cell–gel mixture injection channel); the bottom panel shows representative data of the average velocity (Vx) of DCs in the competing gradients (dark columns: 1.5 mg/mL collagen plus 10% Matrigel; white columns: collagen alone); (**D**) The LN-on-chip model for studying the guidance of CCR7 ligands for T cell migration in LNs. The upper-left panel illustrates the microfluidic device and the method for data analysis; the bottom-left shows a mimicked LN model with complex chemokine gradients; the right panel shows the proposed combinatorial guiding mechanism for T cell trafficking in LN. Figure 5A was adapted from reference [45] with permission from the Royal Society of Chemistry; Figure 5B was adapted from reference [46] with permission from the Royal Society of Chemistry; Figure 5C was adapted from reference [47] with permission from the National Academy of Sciences; Figure 5D was adapted from reference [5] with permission from PLOS.

Dendritic cells (DCs) are the most-potent antigen-presenting cells (APCs) in the immune system [69]. Generally, DCs reside in the peripheral tissues without activation and upon antigen activation they migrate to LNs through lymphatic vessels. In LNs, DCs and T cells interact to enable the secondary immune response [70,71,72]. Both DCs and T cells express the C-C chemokine receptor CCR7, and C-X-C chemokine receptor CXCR4 [73,74]. However, how these receptors and their specific ligands interact to regulate the migration of DCs and T cells and DC–T-cell communication within LNs is not well understood. An in-vitro model that faithfully mimics the LN microenvironment will greatly facilitate understanding DC and T cell migration and their interactions in LNs. In this direction, both 2D and 3D microfluidic models have been developed for immune cell migration studies in LNs (Figure 5; Table 1).

For example, a mimetic LN-on-chip flow device was developed for investigating the interaction between T cells and DCs (Figure 5A) [45]. The device was made of PDMS with one main flow channel connected with two inlets and two outlets, and bonded onto a glass slide. Within the channel, the biomimetic LN tissue was generated by adding different layers: (1) an adsorbed fibronectin (FN) layer; (2) an APC monolayer (which consisted of a DC monolayer and a lipopolysaccharide (LPS) and peptide-major histocompatibility complex (pMHC) layer mimicking chemokine-induced immune response); and (3) a T cell layer. T cells were injected continuously with well-controlled shear stress into the main channel using syringe pumps. At low flow speed (6 μm/min), antigen-specific T cells migrated to the APC monolayer independently, regardless of the flow direction. Moreover, under this low shear condition, stable accumulation of antigen-specific T cells on DCs was observed. Compared to CD8+ T cells, CD4+ T cells showed longer and stronger interactions (attachment and detachment) with APCs under varying shear stress conditions. In addition, a much more stable DC–T cell interaction was found in the presence of specific antigen than unspecific antigen. This LN-on-chip model allowed the investigation of the pMHC–T cell receptor (TCR) bonding mechanism under controlled mechanical force.

In another example, Yarmush and coworkers developed a microfluidic device for evaluating DC chemotaxis and DC–T cell interaction (Figure 5B) [46]. Briefly, the device was made up of two PDMS layers, including the top layer for chemotaxis compartment and the bottom layer for T cell chamber. By recording DC chemotaxis to CCL19 gradient in the chemotaxis compartment, migration behavior of DCs was measured. The DC-promoted T cell activation was evaluated by calculating the calcium level of T cells in the T cell chamber. Based on this LN-on-chip model, mature DCs (mDCs) were shown to cause stronger T cell activation than immature DCs (iDCs). In addition, these results demonstrated the overall T cell activation was mediated by the level of DC migration. This approach allowed systematic investigation of complex immune responses between specific immune cells, such as DC migration and maturation and T cell activation.

In another study, Swartz and coworkers established a 3D agarose-based microfluidic device for investigating differential chemotaxis of DCs to CCR7 ligands CCL21 and CCL19 (Figure 5C) [47]. The device consisted of three main parallel microchannels, including one central channel and two side channels. The central channel was seeded with cell-matrix mixture, while the two side channels were injected with chemokines or buffers, which generated a chemokine gradient in the central channel through agarose. This approach enabled the recapitulation of physiological microenvironment in vitro with specific chemokine gradients, advancing the knowledge of DC homing within LNs. The results showed similar DC migration to both CCL21 and CCL19 gradients at concentrations less than 60 nM. Moreover, DC chemotaxis was enhanced with increasing concentrations of a single gradient of CCL21 or CCL19 with higher migration in CCL21 gradients. In addition, DCs showed stronger migration toward the CCL21 gradient in the presence of a competing CCL19 gradient configured in the opposite direction.

Similarly, Lin and coworkers used a microfluidic device to configure simple or complex co-existing chemical fields for studying the guidance of CCR7 ligands during T cell migration in LNs (Figure 5D) [5]. The “Y”-shaped flow device consisted of two inlets with external perfusion to generate a stable gradient in the main channel. Based on this device, they quantitatively tested the migratory behavior of activated human peripheral blood T cells (aHPBTs) in single CCL19 or CCL21 gradients, and different combinatorial CCL19/CCL21 gradients at physiological doses. The results showed that aHPBTs migrate to the CCL21 gradient alone, but not the CCL19 gradient at the physiological dose. Interestingly, aHPBTs showed repulsive migration from the CCL19 gradient at a low dose with a uniform background of high-dose CCL21, which mimicked the gradient condition at the periphery of the T cell zone (TCZ) in LN. This repulsive migration suggested the role of CCL19 in mediating T cell egress from LNs. Collectively, this model presented an interesting combinatorial guiding mechanism for T cell migration in LNs by CCR7 ligands.

Hammer and coworkers also attempted to reconstitute the complex chemical microenvironment in LNs based on a network-shaped microfluidic flow device for investigating differential DC chemotaxis through CCR7 and CXCR4 signaling [48]. Using this device, well-controlled single and/or competing chemokine gradients were established, which mimicked the complex chemotactic environment in LN tissues. This study showed that CCL19, CCL21 and CXCL12 can potently induce directional DC migration. However, in competing chemokine gradients, CCL19 attracted DCs more effectively than CCL21 or CXCL12, contradicting the results in the 3D microfluidic device, which suggested the different migration and gradient sensing mechanisms in 2D and 3D environments.

## 6. Brain-on-Chip

**Figure 6:**
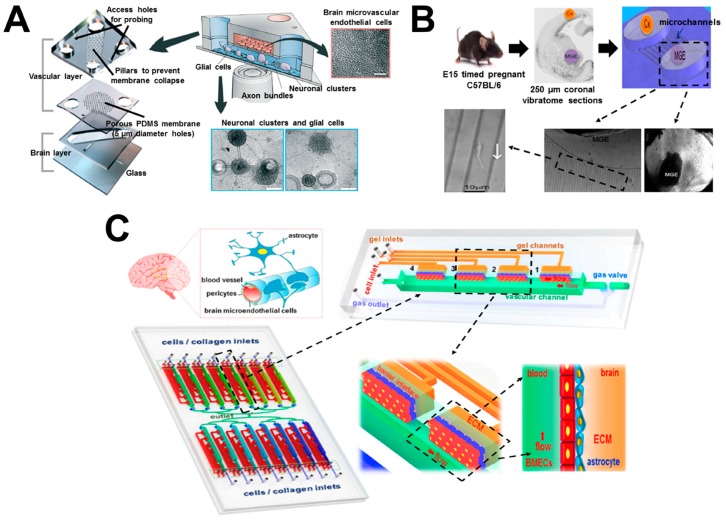
Examples of brain-on-chip cell migration study. (**A**) The brain-on-chip model for investigating neuronal differentiation and chemotaxis. The image shows the detailed information of the microfluidic system. (**B**) The brain-on-chip model for investigating neuronal migration. The upper-left & middle panels show the mouse embryonic brain explants (Cx: cortex; MGE: medial ganglionic eminence); the upper-right panel shows schematic presentation of the microfluidic device; the bottom panel shows representative experimental images of the selective areas. (**C**) The brain-on-chip model for investigating brain tumor metastasis. The image shows the schematic illustration of blood–brain barrier (BBB) and the microfluidic system with magnified views of selective regions. Figure 6A was adapted from reference [49] with permission from the Royal Society of Chemistry; Figure 6B was adapted from reference [50] with permission from Elsevier; Figure 6C was adapted from reference [52] with permission from Nature Publishing Group. BMECs: brain microvascular endothelial cells.

Neural cell migration is an essential process during early embryonic brain development and for the central nervous system (CNS). Neural cell migration is regulated by various chemotactic factors such as inflammatory chemokine CXCL12 [75]. Furthermore, under pathological conditions such as ischemia or tumor growth, CXCL12 can regulate directional migration of neural progenitor cells (NPCs) towards the damaged tissues [76,77]. Therefore, it is important to better understand NPC migration guided by chemical cues in brain tissues, and organ-on-chip offers valuable experimental tools for such studies. Here we review some representative studies in this direction (Figure 6; Table 1).

A recent study generated a biomimetic brain-on-chip model for investigating neuronal differentiation and chemotaxis (Figure 6A) [49]. The device consisted of three PDMS layers. The top compartment was used to culture human brain microvascular endothelial cells (hBMECs) for modeling the blood vessels. The bottom compartment was used to culture glial cells and neuronal clusters for reconstituting the brain parenchyma. The top vascular and the bottom brain layers were separated by an intermediate porous membrane layer, which allowed the interactions of cells from both sides, mimicking the function of the blood–brain barrier (BBB). Using this approach, the differentiation of pluripotent human NTera2 clone D1 (hNT2) cells into mature neuronal and glial cells was achieved. Integration of this neuronal–glial environment with hBMECs enabled the reconstitution of CNS microenvironment in vitro. In addition, tissue-guided chemotaxis of human fetal neural progenitor cells (hNPCs) to different chemokine gradients, including CXCL12 and SLIT2, was also investigated. The results revealed enhanced hNPC chemotaxis in the “brain layer”, indicating the migration of hNPCs in vivo is dependent on the signals in the surrounding CNS tissues.

As another example, Nery and coworkers developed a microfluidic-based method for investigating neuronal migration in embryonic brain explants (Figure 6B) [50]. Briefly, the microfluidic device was made of PDMS, which consisted of two compartments interconnected by multiple capillary microchannels. The compartments and microchannels were injected with gel matrix, mimicking the complex tissue microenvironment in vivo. One mouse medial ganglionic eminence (MGE) explant and one cortical explant were cultured in the two compartments, respectively. Using this brain-on-chip model, individual neuron migration away from the MGE explant to the cortical explant through the capillary microchannels was observed, which demonstrated the long-distance migration of newborn neurons of embryonic MGE to the neocortex for brain development. In addition, this approach allowed observation of the distribution of cellular organelles within migratory neurons in real time, which enabled research to study neuronal migration and its related neurologic diseases.

In another study, Keenan and coworkers established a microfluidic-based method to investigate human neural stem cell (hNSC) neurogenesis [51]. Briefly, the PDMS microfluidic device consisted of two zygomorphic microchannels interconnected by three rectangular and three triangular gradient regions. All the gradient regions were selectively filled with hNSC suspension/Matrigel mixture to mimic the 3D brain ECM environment. The rectangular and triangular gradient regions were responsible for establishing linear and exponential gradients of fibroblast growth factor 2 (FGF-2), respectively. The results of this study demonstrated that the exponential FGF-2 gradient regulated the distribution of hNSCs, and correlated with hNSC neurogenesis, which promotes the development of the cerebral cortex.

In contrast to the above studies that focused on neuronal differentiation and migration, Qin and coworkers developed a mimetic organotypic microfluidic system that reproduced the principal structural, functional and mechanical features of the BBB in vivo, in order to investigate brain tumor metastasis (Figure 6C) [52]. The microfluidic device was made of PDMS, which consisted of 16 independent functional units interconnected by a microchannel network with a shared outlet. Each unit had four individual BBB regions, including one vascular channel for introducing fluidic flow; one gas valve for flow regulation; and four gel channels for loading ECM collagen or astrocytes. This approach reconstituted the key features and functionalities of BBB, including complex cellular interactions, diverse vascular cues, multiple barrier generation, cellular migration and 3D ECM establishment. Furthermore, it enabled real-time observation and analysis of BBB responses during brain tumor metastasis in a high-throughput manner. By using this brain-on-chip model, brain metastasis of human lung, breast and melanoma cells in the BBB microenvironment was investigated. The results demonstrated that the interactions between cancer cells and astrocytes has the potential to promote invasion of malignant tumors to the brain and vascular compartments.

## 7. Conclusions and Outlook

Understanding the mechanisms of cell migration and developing its applications are of great significance for fundamental research and are important targets for disease diagnosis and therapeutics. Organ-on-chip-related approaches offer a promising new concept, enabling technologies to study cell migration in biomimetic tissue or organ microenvironments and to permit clinical applications. These approaches stem from microfluidics and tissue engineering, but the integration of the two in a highly controlled manner is significantly beyond each individual technology or their simple combination. A considerable number of current cell migration studies using organ-on-chip-related approaches employed similar ideas and techniques to reconstitute various organ structures and environments, suggesting the natural emergence of diverse organ-specific applications into the same conceptual and technological framework. We have observed the active research development of this emerging field and we anticipate its continuing growth in the future.

On the other hand, current organ-on-chip technologies for cell migration research are still in their infancy and thus are facing many challenges. Firstly, precise presentation of guiding signals in space and time is an important building block of the organ microenvironment for cell migration. While 3D multi-cell co-culture microfluidic devices more closely mimic physiological tissue or organ microenvironments, the accompanied reduction in their ability to control and manipulate chemical gradients presents a significant drawback compared with 2D systems. In contrast, the 2D microfluidic approach is well established to study cell migration in precisely defined, simple and complex physiologically-relevant chemical fields. While these systems are fundamentally limited to mimic the real in-vivo situations, some cell types evidently show poor or very different migratory behaviors in the 2D systems compared with 3D systems. Therefore, developing organ-on-chip models that effectively integrate the respective advantages of 2D and 3D systems in a balanced manner sets one of the immediate future directions. For example, new techniques must be developed to enable advanced partition of 3D viscous gel zones and soluble flow zones within the microfluidic channels that would allow controlled flow transport over the gel environments. In this direction, 3D bio-printing technology offers a promising approach, owing to its unique ability for controlled and rapid reconstruction of different tissue structures. Recent studies have demonstrated the feasibility of integrating 3D printing with biocompatible micro/nanoparticles for biomimetic bone tissue engineering, which demonstrated the potential of 3D bio-printing for broad organ-on-chip applications [78,79,80]. Similarly, improved techniques are required to better pattern and partition multiple cell types in the device. Another approach can be to embed microcapsules or micro/nanoparticles into the 3D gel-based microfluidic systems to allow controlled chemical release and gradient generation in space and time.

Secondly, while the widely used PDMS microfluidic devices offer advantages in fast prototyping, low cost, biocompatibility and optical transparency, the interactions between various biomolecules and PDMS can be a significant issue for organ-on-chip applications, such as drug testing and ECM configuration. New alternative device fabrication materials are, therefore, being explored. For example, recent studies showed that other elastomers such as polyurethanes offer similar features of PDMS, but higher compatibility with small hydrophobic drugs [81]. Cell migration in microfluidic devices fabricated from these new materials should be tested toward enabling more advanced organ-on-chip applications.

Thirdly, increased complexity of organ-on-chip devices limits experimental throughput compared with simple devices. The throughput issue is also reflected by the isolated, single organ-on-chip approach for many current studies, which underscores the difficulty of accurately mimicking the complex properties of the interacting multi-organ environments in the body, wherein tightly regulated and highly dynamic cell migration and inter-organ trafficking occurs. Thus, innovative and optimized device designs to improve experimental throughput and advanced multi-organs-on-chip devices are highly desirable for future cell migration research. The work of Qin’s group that mimicked the structural, functional and mechanical features of the BBB is an excellent example of such integrated multiple organ-mimicking units with microchannel networks that are required for advanced on-chip cell migration monitoring and analysis [52].

Finally, mimicking disease conditions and testing diseased clinical samples will ultimately enable diagnostic and therapeutic applications of the organ-on-chip approaches. On the other hand, disease-oriented studies are facing significant scientific and technological challenges to faithfully recreate the pathological processes on the chip, which presents an important open question for microfluidics and cell migration researchers to address in the future.

## Figures and Tables

**Table 1:** Summary of cell migration studies using organ-on-chip-related approaches.

Organ Type	Comments	Ref.
**Tumor-on-chip**	Investigating endothelial barrier function during tumor cell intravasation;	[36]
	Investigating immune surveillance of natural killer (NK) cells for tumors;	[37]
	Investigating ECM activation during tumor progression;	[38]
	Investigating the interactions between neurons and cancer cells during tumor perineural invasion.	[39]
**Lung-on-chip**	Investigating bacteria or inflammatory cytokine induced cell migration;	[40]
	Investigating neutrophil chemotaxis with clinical samples for diagnosis of chronic obstructive pulmonary disease (COPD);	[26]
	Rapid analysis of neutrophil chemotaxis;	[41]
	Investigating neutrophil chemotaxis with clinical samples for asthma detection.	[27]
**Vessel-on-chip**	Investigating neutrophil transendothelial migration (TEM) during inflammatory process;	[35]
	Investigating tumor transendothelial invasion (TEI);	[42]
	Investigating tumor cell extravasation;	[43]
	Investigating angiogenesis.	[44]
**Lymph node-on-chip**	Investigating the interaction between T cells and dendritic cells (DCs);	[45]
	Evaluating DC chemotaxis and DC–T cell interaction;	[46]
	Investigating differential chemotaxis of DCs to CCL21 and CCL19;	[47]
	Studying the guidance of CCR7 ligands for T cell migration in Lymph Nodes (LNs);	[5]
	Investigating differential chemotaxis of DCs through CCR7 and CXCR4 signaling.	[48]
**Brain-on-chip**	Investigating neuronal differentiation and chemotaxis;	[49]
	Investigating neuronal migration;	[50]
	Investigating human neural stem cell (hNSC) neurogenesis;	[51]
	Investigating brain tumor metastasis.	[52]

## Abbreviations

3D	three dimensional
ACC	adenoid cystic carcinoma
aHPBTs	activated human peripheral blood T cells
APC	antigen presenting cell
BBB	blood–brain barrier
BME	basement membrane extract
CAF-μTP	cancer–activated fibroblast microtissues
CNS	central nervous system
COPD	chronic obstructive pulmonary disease
DC	dendritic cell
DRG	dorsal root ganglion
EC	endothelial cell
ECM	extracellular matrices
FB	fibroblast
FGF-2	fibroblast growth factor 2
fMLP	*N*-formyl-methionyl-leucyl-phenylalanine
FN	fibronectin
hBMECs	human brain microvascular endothelial cells
HCC	hepatocellular carcinoma
HEV	high endothelial venule
hNPC	human fetal neural progenitor cell
hNSC	human neural stem cell
HUVEC	human umbilical vein endothelial cell
ICAM-1	intercellular adhesion molecule 1
iDC	immature DC
IL-8	interleukin-8
LN	lymph node
LPS	lipopolysaccharide
mDC	mature DC
MGE	medial ganglionic eminence
NF-μTP	normal fibroblast microtissues
NK	natural killer
NPC	neural progenitor cell
PDMS	polydimethylsiloxane
pMHC	peptide-major histocompatibility complex
PNI	perineural invasion
TCR	T cell receptor
TCZ	T cell zone
TEI	transendothelial invasion
TEM	transendothelial migration
TNF-α	tumor necrosis factor alpha
TVM	transvascular migration
μTP	microtissues
μVNs	microvascular networks
